# Hierarchical structural component modeling of microRNA-mRNA integration analysis

**DOI:** 10.1186/s12859-018-2070-0

**Published:** 2018-05-08

**Authors:** Yongkang Kim, Sungyoung Lee, Sungkyoung Choi, Jin-Young Jang, Taesung Park

**Affiliations:** 1 Department of Statistics, Seoul National University, Seoul, Korea; 2 Interdisciplinary program in Bioinformatics, Seoul National University, Seoul, Korea; 3 Department of Surgery and Cancer Research Institute, Seoul National University College of Medicine, Seoul, Korea

**Keywords:** miRNA, mRNA, Integration analysis, Generalized Structured Component Analysis (GSCA), Hierarchical structured component analysis of miRNA-mRNA integration (HisCoM-mimi)

## Abstract

**Background:**

Identification of multi-markers is one of the most challenging issues in personalized medicine era. Nowadays, many different types of omics data are generated from the same subject. Although many methods endeavor to identify candidate markers, for each type of omics data, few or none can facilitate such identification.

**Results:**

It is well known that microRNAs affect phenotypes only indirectly, through regulating mRNA expression and/or protein translation. Toward addressing this issue, we suggest a *hi*erarchical *s*tructured *com*ponent analysis of *mi*croRNA-*m*RNA *i*ntegration (“HisCoM-mimi”) model that accounts for this biological relationship, to efficiently study and identify such integrated markers. In simulation studies, HisCoM-mimi showed the better performance than the other three methods. Also, in real data analysis, HisCoM-mimi successfully identified more gives more informative miRNA-mRNA integration sets relationships for pancreatic ductal adenocarcinoma (PDAC) diagnosis, compared to the other methods.

**Conclusion:**

As exemplified by an application to pancreatic cancer data, our proposed model effectively identified integrated miRNA/target mRNA pairs as markers for early diagnosis, providing a much broader biological interpretation.

## Background

Presently, numerous types of “omics” data are generated by many accurate and cost-effective methods. For instance, next-generation sequencing (NGS) technology is used to find DNA or RNA variations, bisulfite sequencing is used to find DNA-methylated variants, and multiple reaction monitoring (MRM) is applied to measure protein abundances [1–3]. These efficient omics data platforms allow researchers to use multi-omics data, obtained from the same subjects, for analyzing huge numbers of variants. As a result, efficient multi-omics data analysis is becoming more important in integrating large-scale data sets, making it possible to interpret fundamental biological systems [4].

MicroRNAs (miRNAs) are noncoding RNAs having a length less than 25 base pairs, regulating the expression of specific genes by mRNA degradation or blocking translation by binding to the 3′ regions of their “target” mRNAs. Many recent studies have now implicated miRNAs in the pathogenesis of cancer, including triggering cancer initiation and progression. MiRNAs have been shown to have tissue-specific and disease-specific expression patterns [5–8]. Intensive investigation is now underway for using applying miRNAs’ inhibitory information to mRNAs. For example, Nam et al. developed “miRNA and mRNA integrated analysis” (MMIA) to examine biological functions of miRNA expression [9]. Moreover, Buffa et al. used pathway information to independently validate miRNAs significant for breast cancer [10], while Cho et al. performed network analysis, and hierarchical clustering, to find biological “signatures” of interstitial lung diseases [11]. Most miRNA and mRNA integration analyses focus on first identifying miRNAs significantly associated with the phenotype of interest, and then experimentally validating those miRNAs’ phenotype involvement by inhibiting or ectopically overregulating their expression [9–11]. Although these approaches are effective at validating significant miRNAs, they do not provide information on how they regulate expression of their target mRNAs, as relevant to the pathway level.

In this work, we propose a structured component-based analysis, for integrating omics data for identifying multiple accurate biomarkers. It is well known that miRNAs affect phenotypes indirectly, by regulating mRNA expression or protein translation [8]. Herein, we propose **hi**erarchical **s**tructured **com**ponent analysis of **mi**RNA-**m**RNA **i**ntegration (HisCoM-mimi) analysis, which models biological relationships as structured components, to efficiently yield integrated markers. Our proposed model is based on generalized structured component analysis (GSCA), which tests hypothesized relationships between observed and latent variables [12]. GSCA is a component-based method whereby each component represents a latent variable. Extending GSCA, we previously developed Pathway-based approach using hierarchical components of collapsed rare variants (PHARAOH) [13]. PHARAOH uses a hierarchial structure of rare variants, genes, and pathways. The advantage of such hierarchical structural component models is their generation of (unobservable) latent variables, such as genes and pathways, which are inferred by observed variables, such as rare variants. Using latent variables, we can collapse unstructured data into a structured form, providing less ambiguous biological explanations of the results. In this current work, mRNAs, inhibited by miRNAs, can be merged into latent variables.

Accordingly, our proposed HisCoM-mimi model can efficiently account for biological relationships between miRNA and mRNA, in the structured component, and effectively provide integrated (e.g., miRNA-to-target-mRNA) markers. As an illustration, we tried HisCoM-mimi for identifying biomarkers for the early diagnosis of pancreatic cancer (PC). Note that PC is one of the most fatal diseases in the world, having a mere 8% five-year survival rate in the USA and a 9.4% survival rate in the Republic of Korea [14–16]. In particular, the tumor heterogeneity in PC patients’ tumors makes early diagnosis harder than cancers of most other organs [17]. To adjust for heterogeneity among tumor cells, we need a more robust and complex statistical model which can interpret and integrate several causes of cancer altogether. Although many bioinformatics research studies have been performed to find diagnostic markers for PC, to date, no clinically approved prognostic markers exist [18].

Here, we applied HisCoM-mimi to computationally identify diagnostic markers of pancreatic ductal adenocarcinoma (PDAC), the most common type of PC. By applying the HisCoM-mimi approach to miRNA and mRNA microarray data from PDAC patients, at Seoul National University Hospital (SNUH), we identified numerous cognate miRNA-mRNA partners, as markers for diagnosis of PDAC. Finally, our HisCoM-mimi provided integrated marker sets, with more biological and intuitive interpretation, than other existing methods.

## Methods

### Pancreatic ductal adenocarcinoma (PDAC) samples

Between the years 2009 and 2012, 200 pancreatic ductal adenocarcinoma (PDAC) samples were collected by the Department of Hepatobiliary and Pancreas Surgery of Seoul National University Hospital. The study protocol was approved by the Institutional Review Board of Seoul National University Hospital (IRB H-0901-010-267) and written, informed consent was obtained from each patient or legally authorized representative.

Of the 200 tumors, 96 were excluded because of RNA degradation or insufficient RNA content, leaving 104 samples valid for microarray analysis. After quality control, 97 PDAC samples remained for microarray assessment. The PDAC patients’ average age was 64.3 years (standard deviation (SD): 9.7). Twenty-nine patients were male, and 31 female. For the normal groups, 17 benign pancreatic tissues were used. Subsequently, we built and implemented our mini model, using the 97 PDAC and 17 normal tissues, respectively.

### HisCoM-mimi model

**Fig. 1:**
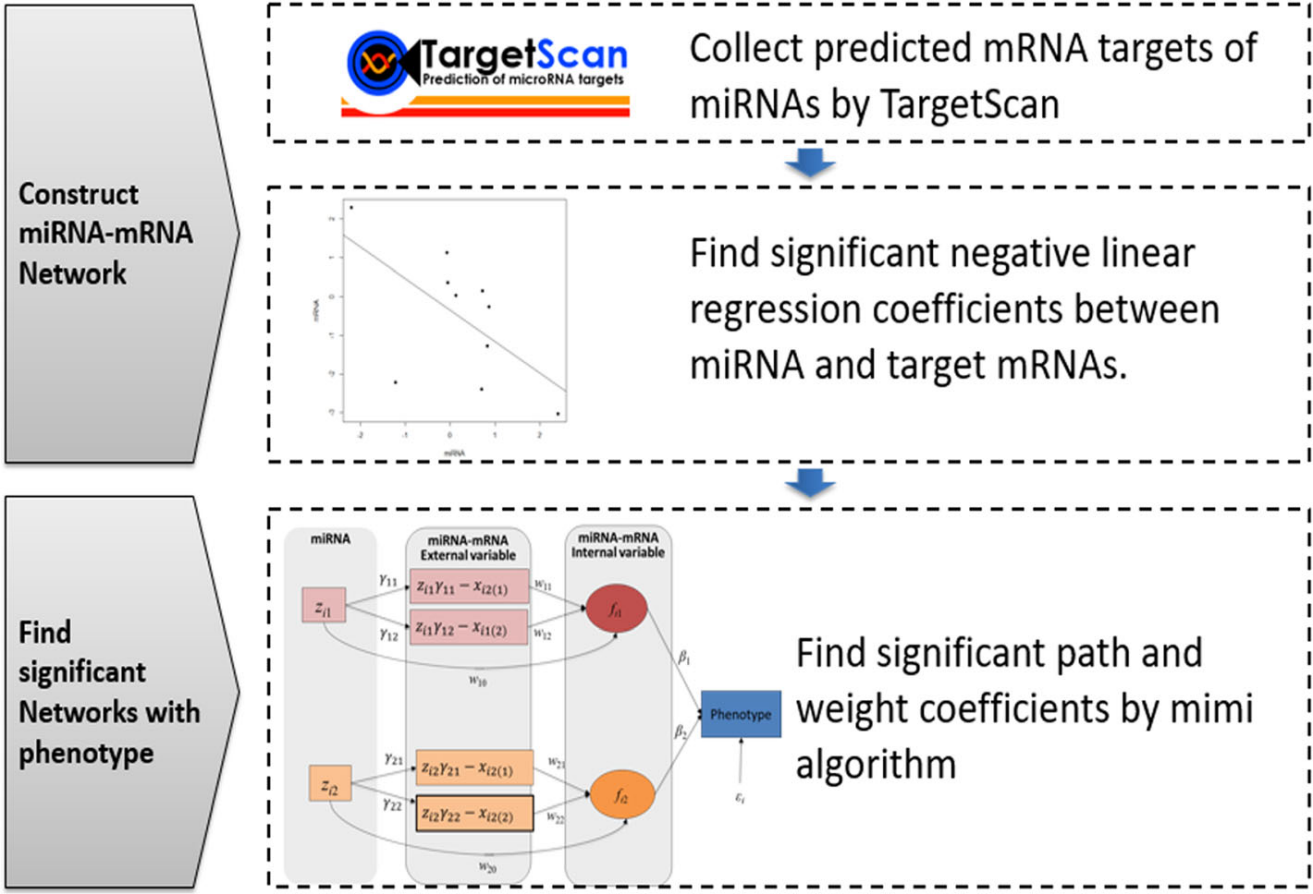
Flow chart for analyzing mRNA-miRNA integration

To perform the integration analysis of miRNA and mRNA data, we developed and implemented our HisCoM-mimi approach. This model analyzes multiple subnetworks simultaneously, with specific regard to inverse correlations between mRNA and miRNA. Figure 1 shows the flowchart of the method. First, for a given miRNA, a miRNA-mRNA subnetwork, consisting of one miRNA and multiple potential target mRNAs, is constructed if the following two conditions are satisfied: (i) the mRNAs are reported as target of the miRNA by TargetScan 7.1 (targetscan.org) [19], and the negative correlation coefficients between the mRNA and miRNAs are significant (*p*-value < 0.05). Second, for all entities deemed significant, we derived our hierarchical structural component model by using all miRNA-mRNA subnetworks.

**Fig. 2:**
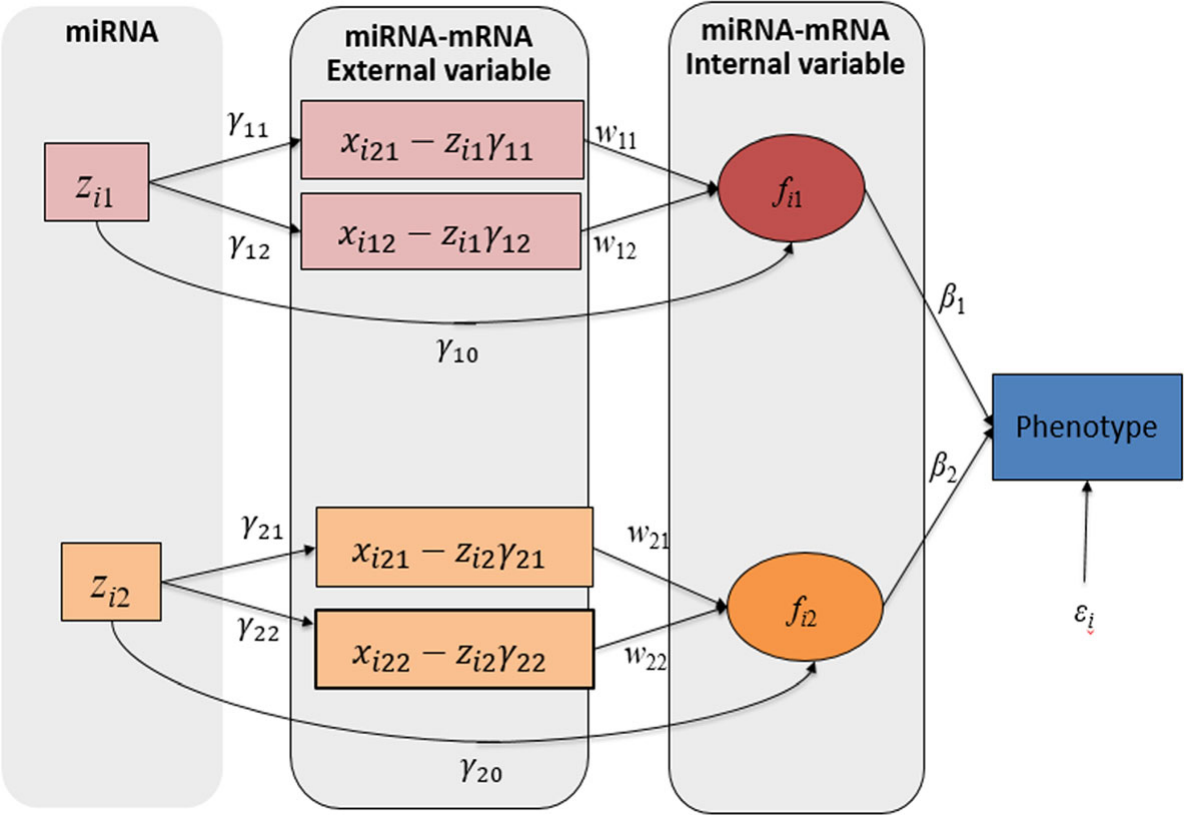
Network Diagram for HisCoM-mimi model

As shown in Fig. 2, there are three structures to consider: miRNA-mRNA structure, miRNA integration latent structure, and phenotype-latent structure. Each structure can be represented as a generalized linear model, similar to PHARAOH [13].

#### miRNA-mRNA structure

1 $$ {\widehat{X}}_{ijk}={x}_{ijk}-{\gamma}_{jk}{z}_{\mathrm{i}j},j=1,\dots, {G}_j, $$

Equation (1) shows how to obtain mRNA expression before inhibition by miRNA, subscript *i* means *i* th individual, *x*_*ijk*_ represents the mRNA expression of the *k*th gene related with *j* th miRNA, *z*_*j*_ the *j* th miRNA expression, *γ*_*jk*_ the inhibition coefficient for the *j* th miRNA for the *k* th gene, and *G*_*j*_ is the number of inhibited mRNAs by the *j* th miRNA. By estimating the coefficients *γ*_*jk*_, mRNA expression after removing the inhibition effect of miRNA can be obtained.

#### miRNA latent structure

2 $$ {f}_{ij}={\gamma}_{j0}{z}_j+{\sum}_{k=1}^{G_j}{\widehat{X}}_{ij k}{w}_{jk} $$

The miRNA latent variable is defined in Eq. (2). The miRNA latent variable is built by linearly combining miRNA expression values. While *γ*_*j*0_ denotes the direct effect of the miRNA on the phenotype. Then, the latent variable *f*_*ij*_ represents the global effect of the miRNA’s activity through its inhibited mRNAs.

#### Phenotype-latent structure

3 $$ logit\left({\pi}_i\right)={\beta}_0+{\sum}_{j=1}^J\left[{\sum}_{k=1}^{G_j}{x}_{ij(k)}^{gene}{w}_{jk}\right]{\beta}_j={\beta}_0+{\sum}_{j=1}^J{f}_{ij}{\beta}_j $$

Let the phenotype variable *y*_*i*_ be a binary variable, distinguishing PDAC from normal tissues. Let *π*_*i*_ be the probability of *y*_*i*_ = 1 (PDAC). *logit*(*π*_*i*_) is the logit link function, *β*_*j*_ represents the effect of *f*_*ij*_ on the phenotype, as interpreted as a log-odds ratio.

#### Fitting the HisCoM-mimi algorithm

To estimate the parameters for HisCoM-mimi, we adopted our previously developed PHARAOH algorithm [13], which is based on the alternating least squares algorithm for the penalized log-likelihood function, with ridge parameters. Then, the objective function to maximize is given as follows:

4 $$ logit\left({\pi}_i\right)={\beta}_0+{\sum}_{j=1}^J\left[{\sum}_{k=1}^{G_j}{x}_{ij(k)}^{gene}{w}_{jk}\right]{\beta}_j={\beta}_0+{\sum}_{j=1}^J{f}_{ij}{\beta}_j, $$

5 $$ {\varphi}_1={\sum}_{i=1}^n\log\ p\left({y}_i;{\beta}_j,\delta \right)-\frac{1}{2}{\lambda}_m{\sum}_{j=1}^J{\sum}_{k=1}^{G_j}{w}_{jk}^2-\frac{1}{2}{\lambda}_{mm}{\sum}_{j=0}^J{\beta}_j^2 $$

where *p*(*y*_*i*_; *γ*_*i*_, *δ*) is the probability distribution for the phenotype of the *ith* individual. *λ*_*m*_ and *λ*_*mm*_ are ridge parameters for miRNA-mRNA pairs of interest, representing the integrated latent components.

To maximize the objective function, *φ*_1_, the iterative reweighted least squares (IRWLS) algorithm is used. Note that when using IRWLS, maximizing *φ*_1_ is equivalent to minimizing the object function *φ*_2_.

6 $$ {\varphi}_2={\sum}_{i=1}^n{\mathrm{v}}_{\mathrm{i}}{\left({z}_i-{\sum}_{j=1}^J{f}_{ij}{\beta}_j\right)}^2-\frac{1}{2}{\lambda}_m{\sum}_{j=1}^J{\sum}_{k=1}^{G_j}{w}_{jk}^2-\frac{1}{2}{\lambda}_{mm}{\sum}_{j=0}^J{\beta}_j^2 $$

### Comparative models

To compare the results of HisCoM-mimi with other methods, we considered several alternative regression-based methods.

7 $$ logit\left({\pi}_i\right)={\beta}_0+{\sum}_{j=1}^J{\theta}_j{z}_{ij}+{\sum}_{k=1}^K{\rho}_k{x}_{ij k},j=1,\dots, J $$

8 $$ {\varphi}_{LR}\left({\beta}_0,\theta, \rho, \delta; X,Z\right)={\sum}_{i=1}^n\log\ p\left({y}_i;{\beta}_0,\theta, \rho \right)-\delta {P}_{\alpha}\left(\theta, \rho \right),j=1,\dots, J $$

Firstly, we considered the ordinary penalized logistic regression (LR) methods such as lasso or elastic-net (EN) [20, 21]. Equation 7 shows the LR model, where *θ*_*j*_ and *ρ*_*k*_ represent the effect of the *j*th miRNA and the *k*th mRNA, respectively. Equation 8 is the objective function to maximize for finding optimal parameters with the penalty function *P*_*α*_(*θ*, *ρ*). When lasso is used, *P*_*α*_(*θ*, *ρ*) =∑_k_ ∣ *ρ*_*k*_ ∣  + ∑_j_ ∣ *θ*_*j*_∣.

If EN is used, ${P}_{\alpha}\left(\theta, \rho \right)=\alpha \left({\sum}_{\mathrm{k}}\left|{\rho}_k\right|+{\sum}_{\mathrm{j}}\left|{\theta}_j\right|\right)+\left(1-\alpha \right)\left({\sum}_{\mathrm{k}}{\rho}_k^2+{\sum}_j{\theta}_j^2\right)$. Lasso or EN can then select the miRNAs and/or mRNAs of interest. However, these methods cannot use group information. Thus, ordinarily penalized LR methods cannot adequately account for the biological structure of miRNA-mRNA.

Secondly, we considered LR with a group lasso penalty (GL) [22], which has the benefit of using group information among the miRNAs and mRNAs of interest. In our analysis, a group can be defined as a set of one miRNA and its corresponding inhibited target mRNAs. GL uses the same LR in (8) with a different penalty function $P\left(\theta, \rho \right)={\sum}_{j=1}^J\sqrt{\theta_j^2+{\sum}_{k=1}^{G_j}\left|{\rho}_k\right|}$. Via this penalty function, miRNA integration set can be selected together. However, the GL approach does not easily provide *p*-values for each set of independent variables.

To fit the penalized LR models, we first performed 3-fold cross-validation to find the optimal tuning parameter, δ. after which we fitted the models with all the data sets.

### Simulation study

To compare HisCoM-mimi to the other three methods, we performed simulation studies and computed type I errors and power, simulating data from the same miRNA and mRNA data structure in our pancreatic cancer dataset. That is, we selected miRNA and mRNA data from the pancreatic cancer dataset, and then generated phenotype data iteratively from the LR model. We then considered two simulation scenarios. Scenario 1 assumed that a true causal integration set contains two mRNAs, with the same effect size. Scenario 2 assumed that a true causal integration set contains five mRNAs, with the same effect size. For each scenario, we randomly selected one causal miRNA-mRNA subnetwork, and then randomly selected another 9 miRNA-mRNA subnetworks, for which the number of inhibited mRNAs was less than 10. The selected miRNA-mRNA subnetworks for Scenario 1 are summarized in Table 1 and for Scenario 2 are in Table 2.

**Table 1:** List of used miRNAs and mRNAs for simulation Scenario 1

miRNA	Role in simulation	Inhibited mRNA
miR-217	Causal	ITGBL1, ATP10A
miR-215	Non-Causal	CDC6, CTH, DNAJC19, DPP10, ELP4, FUNDC2, GLP1R, B3GALNT2, SLC39A8
miR-485	Non-Causal	CDX1, CTDNEP1, GPR3, HDAC5, KCNJ11, RASL10A, SLC39A14
miR-195	Non-Causal	CNDP2, SLC45A2, SLC7A2
miR-381	Non-Causal	DKK3, IGFBP5, LAMA4, OSBPL3, BAMBI
miR-132	Non-Causal	GLRB, GMPR, ARX, SALL3
miR-363	Non-Causal	SOSTDC1
miR-1	Non-Causal	FAM150B
miR-28	Non-Causal	SRPRB
miR-200	Non-Causal	NRG3

**Table 2:** List of used miRNAs and mRNAs for simulation Scenario 2

miRNA	Role in simulation	inhibited mRNA
miR-381	Causal	DKK3, IGFBP5, LAMA4, OSBPL3, BAMBI
miR-215	Non-Causal	CDC6, CTH, DNAJC19, DPP10, ELP4, FUNDC2, GLP1, B3GALNT2, SLC39A8
miR-32	Non-Causal	COL1A2, BGN
miR-195	Non-Causal	CNDP2, SLC45A2, SLC7A2
miR-501	Non-Causal	PARM1, SLC32A1
miR-1	Non-Causal	FAM150B
miR-212	Non-Causal	KCNK2
miR-204	Non-Causal	CDH11
miR-200	Non-Causal	NRG3
miR-363	Non-Causal	SOSTDC1

For Scenario 1, we used miR-217 as a true causal miRNA. To generate phenotypes, we considered the following LR model.

9 $$ logit\left(\pi \right)={\beta}_{miRNA}{z}_1+{\beta}_1{x}_1+{\beta}_2{x}_2, $$

where *π* is the probability of observing a disease (*Y* = 1), *z*_1_ represents the true causal miRNA expression, and *x*_1_  and *x*_2_ represent two causal mRNA expression values. For type I error evaluation, we assumed *β*_*miRNA*_ = *β*_1_ = *β*_2_ = 0. For power comparison, we generated simulation data sets under the assumption that *β*_*miRNA*_ = *β*_1_ = 0.2,  0.25, 0.3, 0.35. For the given 114 (97 PDAC and 17 normal tissues) values of (*z*_1_, *x*_1_,  *x*_2_), from our pancreatic cancer dataset, we simulated 1000 datasets.

For Scenario 2, we assumed that a true causal integration set contains five mRNAs, with the same effect size. In our dataset, miR-381 was the only miRNA having five inhibited target mRNAs. To generate phenotypes, we considered the following LR model:

10 $$ logit\left(\pi \right)={\beta}_{miRNA}{z}_1+{\beta}_1{x}_1+{\beta}_2{x}_2+{\beta}_3{x}_3+{\beta}_4{x}_4+{\beta}_5{x}_5, $$

where *x*_1_, …, *x*_5_ represent five causal mRNA expression values. As in Scenario 1, we assumed *β*_*miRNA*_ = *β*_1_ = *β*_2_ = *β*_3_ = *β*_4_ = *β*_5_ = 0, for type I error evaluation, and *β*_*miRNA*_ = *β*_1_ = *β*_2_ = *β*_3_ = *β*_4_ = *β*_5_ = 0.2, 0.25, 0.3, 0.35, for power comparison. For the given 114 values of (*z*_1_, *x*_1_,  *x*_2_, *x*_3_,  *x*_4_, *x*_5_) from the pancreatic cancer dataset, 1000 simulation datasets were generated. We used the significance level α = 0.05 for HisCoM-mimi, as an false positive rate (FPR) criterion. For lasso, EN, and group-lasso, we selected a threshold *T* which provides a comparable FPR to the type I error 0.05. *T* was determined by calculating the FPR for simulation settings such that a miRNA-mRNA subnetwork is selected when *β*_*miRNA*_ ≠ 0 and $K\left(={\sum}_{\mathrm{l}=1}^{\mathrm{L}}I\left({\beta}_l\ne 0\right)\right)$ exceeded the threshold *T*. Here, *L* is the number of inhibited mRNAs for true causal miRNA for each scenario: *L* = 2 for Scenario 1, and *L* = 5 for Scenario 2.

## Results

### Simulation results

For our analyses, we first determined the false positive error rates (FPRs) of each method, and chose the threshold values of T to make each penalized method provide (hold) FPRs close to 0.05. In Scenario 1, the type I error rate of HisCoM-mimi was 0.048 when α = 0.05. The FPRs of lasso were 0.054, when T was 1, and that of EN was 0.064, when T was 1. Since type I error rates of lasso and EN were nearly 0.05 when T = 1, we set T = 1 to evaluate power of those two methods. The FPR of GL, when choosing a causal miRNA integration set, 0.064.

**Fig. 3:**
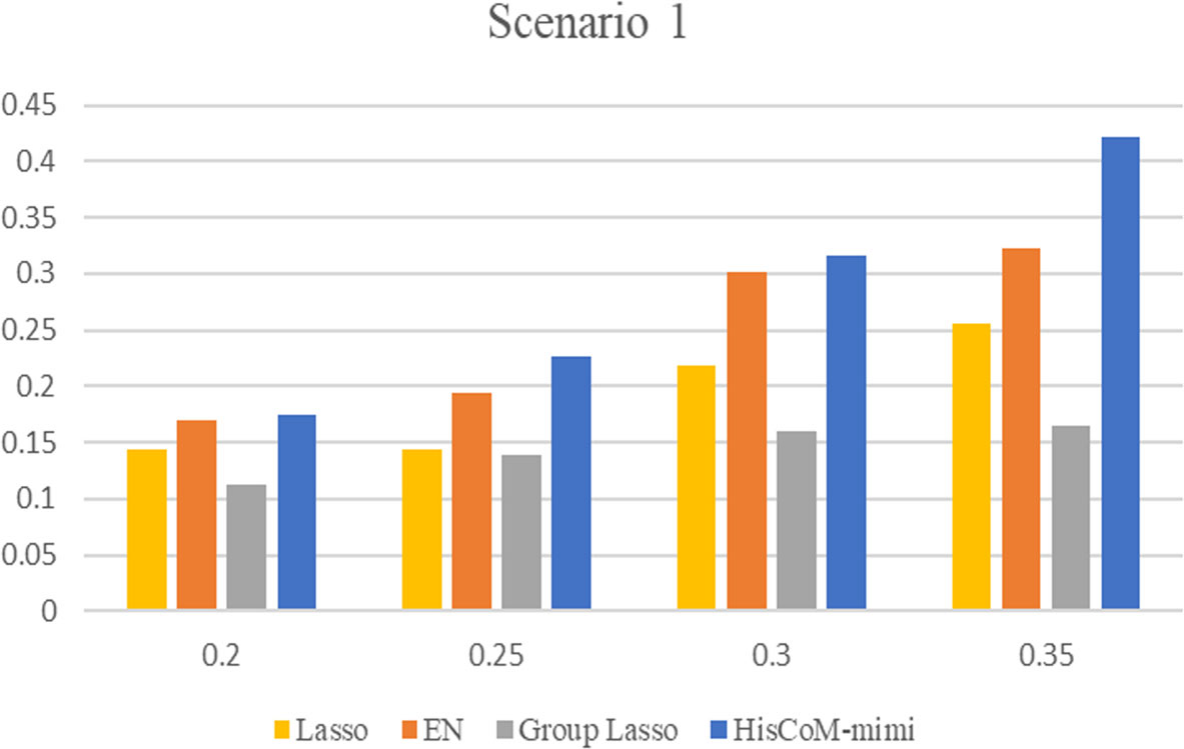
Power comparison for scenario 1

**Fig. 4:**
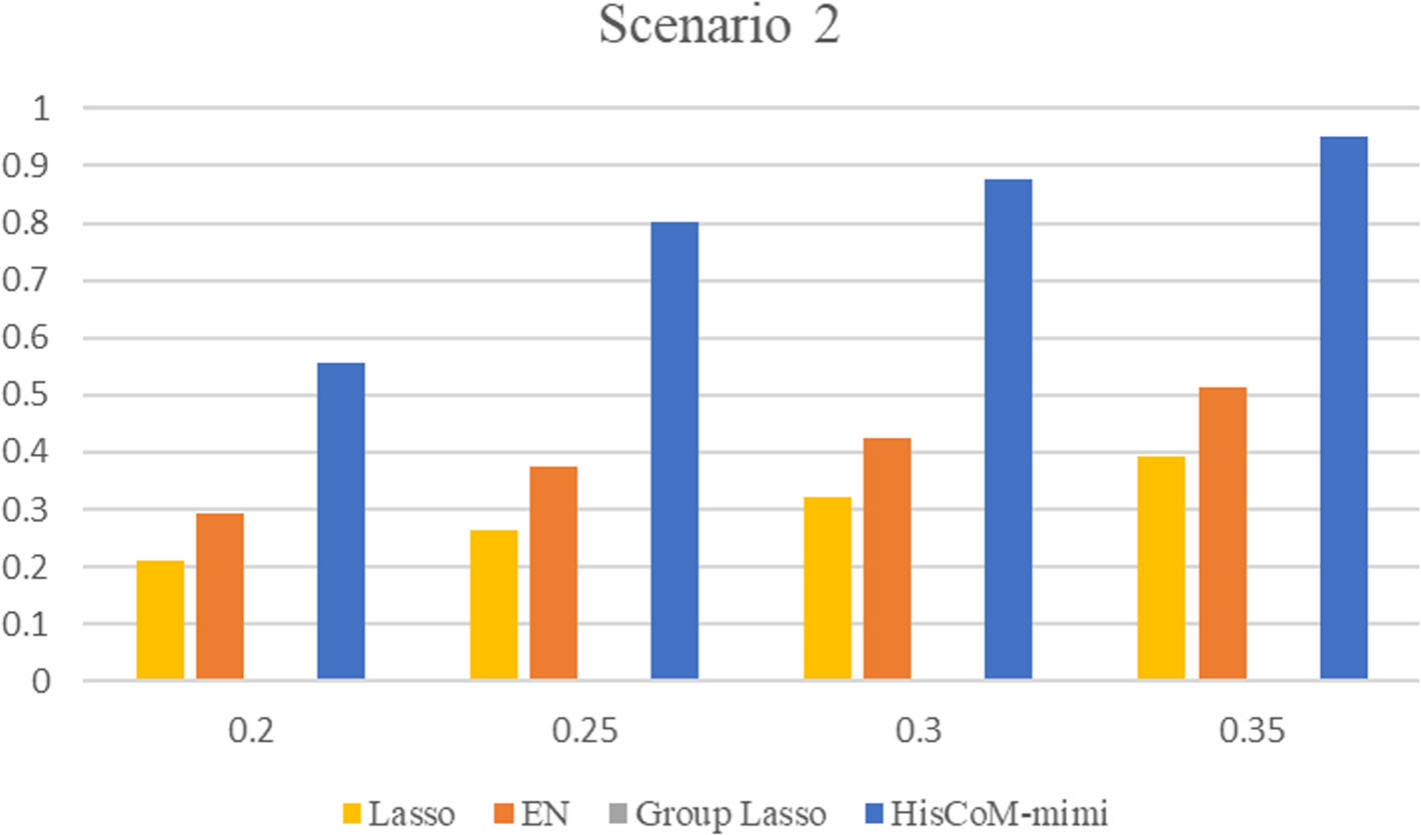
Power comparison for scenario 2

**Fig. 5:**
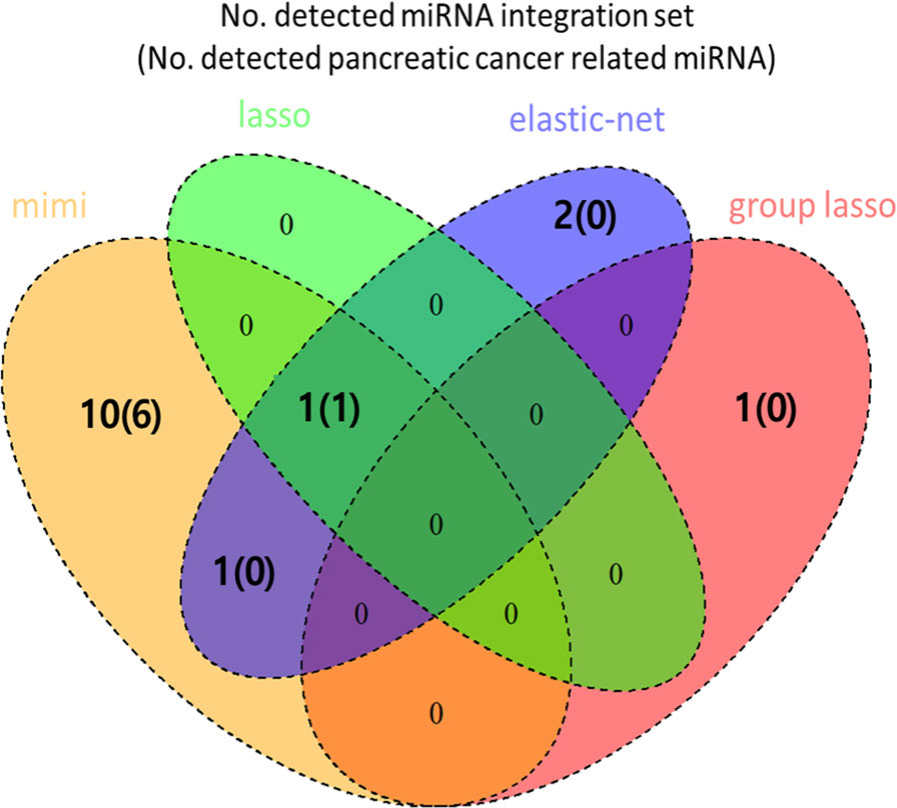
Venn Diagram for number of detected miRNAs for each method

For Scenario 2, Table 3 shows the FPRs for lasso and EN, when varying the threshold T. For this result, we found that the type I error of lasso and EN were similar to 0.05, when T = 1 and 2, respectively. The type I error rate of HisCoM-mimi was 0.054. On the other hand, GL did not select a causal miRNA integration set at all, such that the type I error rate was 0. Secondly, we compared the powers of each method for Scenarios 1 and 2. Figure 3 shows bar plots of powers for scenario 1, where the x-axis shows the effect sizes (i.e., beta coefficients), and the y-axis shows the power. HisCoM-mimi showed the highest power, while EN was second, Lasso was third, and GL was last. The same tendency is shown in Fig. 4, for Scenario 2. Figure 5 shows that the differences of power between HisCoM-mimi and the others were much larger than those of Scenario 1. Consequently, GL could not find any significant miRNA-mRNA integration sets under Scenario 1, due to its GL’s penalty being too strict for many mRNAs, whose beta values were small.

**Table 3:** False positive rate when varying the number of selected mRNAs for lasso and EN

T	5	4	3	2	1
Lasso	0	0	0.007	0.022	0.053
EN	0	0.002	0.014	0.055	0.204

### Constructing miRNA-mRNA subnetworks

To use human mRNA and miRNA probes, we first filtered out non-annotated mRNA probes and non-human miRNA probes. After filtering, there were 22,077 mRNA probes and 3391 miRNA probes. To construct miRNA-mRNA subnetworks, we checked predicted target mRNAs, for each miRNA, from TargetScan 7.1 (targetscan.org) [19, 23]. Among predicted targets, we only selected mRNAs having significant Pearson correlation coefficients with a specific miRNA. After filtering, there were 55 miRNAs, and 2411 edges connected with mRNAs.

### Integration analysis for the PDAC data

Table 4 shows the top significant weights of miRNA-mRNA integrations derived from HisCoM-mimi. To perform multiple comparison, we used false discovery rate (FDR) q-values summarized in the 7th column [24]. We could only find 12 miRNAs having q-values below 0.05. Tables 5 and 6 show the lists of the selected markers by lasso and EN, respectively. Since lasso and EN select markers without any group information, they selected miRNA and mRNA markers independently. There were no miRNAs selected by lasso or EN directly, with lasso yielding only two significant mRNAs, both related to miR-326. Other mRNAs were independently selected from different miRNAs. Consequently, there were only 12 markers selected by lasso. For EN, 58 mRNAs were selected. Similar to the lasso result, there were no selected miRNAs, although four miRNAs (miR-206, miR-3064, miR-222, and miR-326) connected to more than three mRNAs. Figure 5 shows a Venn diagram of the number of miRNAs selected by each method. Each number represents the total number of detected miRNAs and one in the parenthesis does the number of detected miRNAs whose relationship with pancreatic cancer were reported. HisCoM-mimi selected larger number of unique miRNAs and the majority of them were already were reported.

**Table 4:** Significant miRNAs produced by HisCoM-mimi

Order	miRNA	Number of inhibited mRNAs	Number of significant mRNAs	***β***_***mimi***_	*P*_*HisCoM-mimi*_	*q*_*HisCoM-mimi*_
1	miR-133b	81	29	0.319	0.0008	0.0126
2	miR-141	105	57	0.638	0.0008	0.0126
3	miR-222	127	70	0.587	0.0010	0.0126
4	miR-532	11	0	0.190	0.0010	0.0126
5	miR-93	80	36	−0.573	0.0014	0.0126
6	miR-219	26	3	0.278	0.0016	0.0126
7	miR-590	24	4	−0.183	0.0016	0.0126
8	miR-326	13	0	0.172	0.0022	0.0151
9	miR-203	65	11	−0.261	0.0026	0.0159
10	miR-132	4	0	−0.204	0.0034	0.0187
11	miR-96	109	42	0.701	0.0038	0.0190
12	miR-708	43	3	−0.181	0.0102	0.0468

**Table 5:** Selected markers by lasso. Twelve markers (12 mRNAs) were selected. No miRNAs were selected

Selected marker	Beta	Connected miRNA	Selected marker	Beta	Connected miRNA
NSD1	− 0.704	miR-206	PLCE1	0.129	miR-1271
EMX2	−0.336	miR-222	TFCP2	0.112	miR-497
BBC3	0.329	miR-222	AKAP7	−0.017	miR-1297
GSG1	0.005	miR-3064	MAMDC2	1.044	miR-670
ZRANB3	−0.414	miR-326	DRGX	0.393	miR-96
MLEC	0.051	miR-362	FBXL2	−0.187	miR-133b

**Table 6:** Markers selected by EN

Selected mRNA	Beta	Connected miRNA	Selected mRNA	Beta	Connected miRNA	Selected mRNA	Beta	Connected miRNA
NSD1	− 0.340	miR-206	NUP214	−0.103	miR-3064	TFCP2	0.216	miR-497
FRS2	−0.046	miR-206	TCP11	−0.077	miR-3064	KDM5B	0.040	miR-524
MGAT4A	0.004	miR-206	BCL2L13	−0.022	miR-3064	RNASEH2C	−0.043	miR-670
SLC8A1	0.022	miR-206	SLC16A10	−0.016	miR-3064	MAP3K10	0.163	miR-670
PI4KA	0.027	miR-206	GSG1	0.034	miR-3064	MAMDC2	0.395	miR-670
MATR3	0.034	miR-206	LRRC34	−0.159	miR-326	TCEB3	−0.286	miR-93
OSBPL8	0.088	miR-206	ZRANB3	−0.127	miR-326	RASL11B	0.036	miR-93
EMX2	−0.275	miR-222	AQP2	−0.037	miR-326	KIAA0087	0.182	miR-96
KIAA0430	−0.039	miR-222	CTRC	−0.007	miR-326	DRGX	0.249	miR-96
AXIN2	0.003	miR-222	MLEC	0.034	miR-362	HS3ST2	0.016	miR-100
PRUNE	0.013	miR-222	NOTCH1	0.003	miR-367	SYDE2	0.098	miR-107
SHISA9	0.016	miR-222	SH3PXD2A	0.014	miR-367	AKAP7	−0.207	miR-1297
SHC3	0.031	miR-222	PTDSS1	0.017	miR-372	FBXL2	−0.373	miR-133b
RBL1	0.044	miR-222	CATSPER4	0.002	miR-378	CLIP2	0.005	miR-141
SOCS1	0.053	miR-222	TRIM55	0.071	miR-378	LYPD3	0.188	miR-152
SH3BP4	0.057	miR-222	SLC35E2B	−0.128	miR-488	PAQR9	0.308	miR-152
BBC3	0.074	miR-222	SALL4	−0.080	miR-1271	SCN1A	0.017	miR-203
SEC23IP	0.077	miR-222	MAGI3	0.009	miR-1271	CCPG1	0.070	miR-211
ESR1	0.085	miR-222	PLCE1	0.198	miR-1271	BGN	−0.161	miR-32
DGKI	−0.003	miR-330-5p						

For the lasso group only one miRNA (miR-32) and whose related two mRNA (COL1A2, and BGN) were selected. Although miR-32 is not reported as pancreatic cancer marker, there were some reports that miR-32 is related with other cancers [25, 26].

Table 7 summarizes miRNAs detected by HisCoM-mimi, lasso, EN, or GL. Previously, miR-93, miR-219, miR-141, miR-222, miR-203, miR-132, miR-96, and miR-206 were reported to be pancreatic cancer-related markers [27–35]. Although other miRNAs detected by HisCoM-mimi, lasso, EN, or GL have not been reported for pancreatic cancer relation, miR-532, miR-590, miR-133b, miR-326, miR-708, miR-3064, and miR-32 were reported to associate with other cancer types [25, 36–42].

**Table 7:** Cancer related miRNAs detected by methods

Method	miRNA	Number of used mRNA	Reported cancer relationship	Method	miRNA	Number of used mRNA	Reported cancer relationship
HisCoM-mimi	miR-93	80	Pancreas	HisCoM-mimi	miR-132	4	Pancreas
HisCoM-mimi	miR-219	26	Pancreas	HisCoM-mimi	miR-96	109	Pancreas
HisCoM-mimi	miR-532	11	Other	HisCoM-mimi	miR-708	43	Other
HisCoM-mimi	miR-590	24	Other	Lasso	miR-222	2	Pancreas
HisCoM-mimi	miR-141	105	Pancreas	EN	miR-206	7	Pancreas
HisCoM-mimi	miR-133b	81	Other	EN	miR-222	12	Pancreas
HisCoM-mimi	miR-222	127	Pancreas	EN	miR-3064	5	Other
HisCoM-mimi	miR-203	65	Pancreas	EN	miR-326	4	Other
HisCoM-mimi	miR-326	13	Other cancer	GL	miR-32	2	Other

Table 8 shows the cross-validation (CV) results for comparing prediction performance for marker-sets selected by HisCoM-mimi, Lasso, EN, and Group Lasso. The first column indicates methods used to construct prediction model and the second column does the method to select marker sets. The third column shows the area under the Receiver Operating Characteristic curve (AUC) results performed by leave-one-out cross validation (LOOCV). This setting is from the previous study of Kwon et al. [23]. The fourth column indicates the average AUC values performed by four-fold CV with a hundred iterations. Here, we used four-fold and eight-fold CV to balance the number of samples in CV datasets. The fifth column indicates the average AUC values performed by eight-fold CV with a hundred iterations. For all selected marker-sets, all prediction models built by HisCoM-mimi showed the best performances yielding AUC values higher than 0.9 except the marker-set selected by Group lasso in which the number of markers is less than five and one path coefficient exists.

**Table 8:** Evaluation of Prediction performance for marker set selected by HisCoM-mimi, Lasso, EN, or Group Lasso in PDAC samples

Marker set	Method	AUC-loocv	AUC-4-fold CV	AUC-8-fold CV
HisCoM-mimi	HisCoM-mimi	**0.997**	**0.996**	**0.997**
	Lasso	0.948	0.947	0.948
	EN	0.975	0.969	0.971
	Group Lasso	0.889	0.888	0.895
Lasso	HisCoM-mimi	0.976	0.975	0.976
	Lasso	0.938	0.928	0.939
	EN	0.970	0.953	0.963
	Group Lasso	0.910	0.910	0.918
EN	HisCoM-mimi	0.976	0.976	0.976
	Lasso	0.939	0.927	0.935
	EN	0.969	0.957	0.965
	Group Lasso	0.911	0.912	0.915

## Discussion and conclusion

In this paper, we proposed and developed a novel method, **hi**erarchical **s**tructured **com**ponent analysis of **mi**croRNA-**m**RNA **i**ntegration (“HisCoM-mimi”), to construct a component model to identifying significantly integrated miRNA-target-mRNA cognate pairs. Since HisCoM-mimi could use subgroup information, it yelded more results, as related to phenotypes (e.g. cancer, metabolic syndrome, and etc.), than those of other existing methods that lack network information.

In simulation studies, we compared the performances of HisCoM-mimi, lasso, EN, and GL. From that comparison, HisCoM-mimi showed better performance than the other three methods. Controlling type I error, by HisCoM-mimi, was easier for controlling FPRs than other methods, because HisCoM-mimi uses permutation based *p*-values. In particular, HisCoM-mimi could identify miRNA-mRNA integration sets in a much more flexible way, due to better use of a standard multiple testing framework, as compared to the other methods. In real data analysis, HisCoM-mimi succesfully identified more miRNA-mRNA integration sets for pancreatic ductal adenocarcinoma (PDAC) diagnosis, compared to the other methods. Among 12 miRNAs, whose q-values were below 0.05 by HisCoM-mimi, 7 miRNAs were previously reported to associate with a panreatic cancer [27–35]. EN found two miRNAs (miR-222, and miR-206) [30, 34]**.** Among two miRNAs selected by lasso, only miR-222 was reported to associate with pancreatic cancer.

Although HisCoM-mimi worked well for the PDAC data sets, further biological verification of those results are needed. In future studies, we will perform additional simulation analyses to evaluate the performance of HisCoM-mimi, under numerous conditions. Furthermore, HisCoM-mimi can be extended in many ways, for other types of phenotypes, such as time to event. Second, it can be easily applied to other cancer studies to identify miRNA-mRNA integration sets for early diagnosis and prognosis. Third, it can be extended to combine other types of omics data such as genomics, epignomics, and proteomics data. It is now established that dysregulated miRNAs play substantial roles in a myriad of diseases [43]. We firmly believe that these methods for miRNA identification and their target transcripts could yield effective biomarkers and therapeutic targets, in addition to providing better understanding of disease mechanisms and etiology.

## Abbreviations

AUC: Area under the receiver operating characteristic curve
CV: Cross-validation
EN: Elastic-net
FPR: False positive rate
GL: Group lasso
GSCA: Generalized structured component analysis
HisCoM-mimi: Hierarchical structured component analysis of microRNA-mRNA integration
IRWLS: Iterative reweighted least squares
JJ: Jin-Young Jang
LR: Logistic regression
PDAC: Pancreatic ductal adenocarcinoma
PHARAOH: Pathway-based approach using hierarchical components of collapsed rare variants
SC: Sungkyoung Choi
SL: Sungyoung Lee
SNUH: Seoul National University Hospital
TP: Taesung Park
YK: Yongkang Kim
